# High-throughput and genome-guided optimization of exopolysaccharide production in marine bacteria for sustainable biotechnology

**DOI:** 10.1128/aem.00837-25

**Published:** 2025-10-21

**Authors:** Yajun Wei, Fang Zhou, Zhangjian Feng, Min Qi, Rong Xiang, Hongbo Yi, Qiao Yang, Xi Yang

**Affiliations:** 1 Fuyao University of Science and Technology 672513 , Fuzhou, Fujian, China; 2 State Key Laboratory of Swine and Poultry Breeding Industry, Key Laboratory of Animal Nutrition and Feed Science in South China, Ministry of Agriculture and Rural Affairs, Guangdong Provincial Key Laboratory of Animal Breeding and Nutrition, Institute of Animal Science, Guangdong Academy of Agricultural Sciences 117866, Guangzhou, China; 3 Sericultural and Agri-Food Research Institute, Guangdong Academy of Agricultural Sciences, Key Laboratory of Functional Foods, Ministry of Agriculture and Rural Affairs, Guangdong Key Laboratory of Agricultural Products Processing 117866, Guangzhou, China; 4 Institute of Animal Health, Guangdong Academy of Agricultural Sciences 117866, Guangzhou, China; 5 ABI Group, Phycosphere Microbiology Laboratory, Zhejiang Ocean University 71233, Zhoushan, China; Danmarks Tekniske Universitet The Novo Nordisk Foundation Center for Biosustainability, Kgs. Lyngby, Denmark

**Keywords:** marine bacteria, exopolysaccharides (EPS), sustainable bioprocessing, genomic-guided optimization, high-throughput fermentation, circular bioeconomy, industrial EPS production

## Abstract

Marine bacteria are an untapped resource for exopolysaccharides (EPS) with broad potential applications in biomedicine, bioremediation, and food industries. However, industrial utilization remains limited due to low yields and strain-specific variability. This study explores seven novel marine bacterial strains (*Limnobacter alexandrii* LZ-4, *Nioella ostreopsis* Z7-4, *Mesorhizobium alexandrii* Z1-4, *Marinobacter shengliensis subsp. Alexandrii* LZ-6, *Marinobacter alexandrii* LZ-8, *Memelialla alexandrii* LZ-28, and *Sulfitobacter alexandrii* AM1-D1) isolated from marine dinoflagellate microbiota. A genome-guided, high-throughput fermentation strategy was employed to optimize EPS production. Genomic analysis identified distinct EPS biosynthesis pathways (e.g., alginate and cellulose synthesis) and key genes (*algA/C/D, bcsB,* and *epsE/H/J*) involved in both the polymerization and the secretion of EPS. High-throughput screening under 50 fermentation conditions revealed sucrose and fructose as optimal carbon sources, with alkaline pH (7–9) significantly enhancing EPS yields (up to 159.6 µg/mL). Strain-specific optimization demonstrated that LZ-4 and Z7-4 achieved maximal EPS production at 28°C, whereas LZ-8 exhibited a high EPS production at 37°C. The study underscores the synergy between genomic insights and systematic screening, offering a scalable framework for rapid strain optimization. These findings pave the way for sustainable EPS bioproduction, reducing reliance on synthetic polymers and advancing industrial biotechnology in alignment with circular bioeconomy goals.

**IMPORTANCE:**

This study integrates genomic analysis with high-throughput fermentation to optimize exopolysaccharide (EPS) production in seven novel marine bacterial strains. By identifying key EPS biosynthesis genes and pathways, we tailored fermentation conditions using sucrose and alkaline pH, achieving yields up to 159.6 µg/mL. Strain-specific optimizations revealed significant enhancements in EPS production, highlighting the potential for sustainable industrial applications. This work bridges ecological insights with bioprocessing, offering a scalable framework for efficient EPS production that reduces reliance on synthetic polymers, advancing circular bioeconomy goals. The findings underscore the importance of marine microbial resources in biotechnology.

## INTRODUCTION

Exopolysaccharides (EPS) produced by marine bacteria have emerged as versatile biomaterials with applications in bioremediation, pharmaceuticals, and functional food additives. Their unique rheological, antioxidant, and metal-binding properties make them highly valuable in industrial and environmental contexts (1, 2). Among these, marine phytoplankton-associated bacteria represent an underexplored reservoir of EPS diversity. The symbiotic interactions between these bacteria and phytoplankton likely drive the evolution of specialized metabolic pathways, offering a unique opportunity for industrial biotechnology (3, 4).

Despite their potential, the industrial adoption of microbial EPS remains constrained by low yields (typically <200 mg/L for most reported strains) and the labor-intensive nature of optimization processes (5, 6). Overcoming these challenges is essential to unlock the potential of marine EPS as a sustainable alternative to synthetic polymers in high-value industries. Traditional fermentation optimization methods, such as one-factor-at-a-time (OFAT) approaches, often fail to account for complex interactions among parameters and strain-specific metabolic heterogeneity, limiting their effectiveness (7). Furthermore, although genomic predictions of EPS biosynthesis pathways have advanced significantly, they often lack experimental validation, creating a critical gap in translating sequence data into scalable bioprocesses (8).

Recent advances in genomics and high-throughput screening (HTS) provide new tools to address these limitations. Genome mining has enabled the prediction of EPS biosynthesis capabilities, such as alginate, cellulose, or hyaluronan pathways, whereas HTS allows for rapid evaluation of strain performance under diverse conditions (9, 10). However, few studies have systematically integrated these approaches, particularly for newly discovered marine bacterial species (11).

To address these challenges, this study employed a dual-pronged approach combining genomic annotation and HTS to optimize EPS production in seven novel marine bacterial species isolated from marine dinoflagellate microbiota. Specifically, this study aims to (i) identify strain-specific EPS biosynthesis genes (such as alg, bcs, and eps clusters) that are associated with the characteristics of the produced polymers; (ii) determine the optimal combinations of carbon sources, pH, and temperature through high-throughput fermentation experiments; and (iii) evaluate the correlations between genomic predictions and experimentally measured EPS yields.

By bridging ecological insights with industrial bioprocessing, this work establishes a scalable framework for marine EPS exploitation. It also advances our understanding of phytoplankton-bacteria symbiosis, providing a foundation for sustainable biopolymer production in line with global efforts toward circular bioeconomies (12, 13).

## MATERIALS AND METHODS

### Bacterial strains and isolation

Seven novel bacterial strains (*Limnobacter alexandrii* LZ-4, *Nioella ostreopsis* Z7-4, *Mesorhizobium alexandrii* Z1-4, *Marinobacter shengliensis subsp. alexandrii* LZ-6, *Marinobacter alexandrii* LZ-8, *Memelialla alexandrii* LZ-28, and *Sulfitobacter alexandrii* AM1-D1) were isolated from the surface microbiota of marine dinoflagellates (*Alexandrium minutum* and *Prorocentrum lima*) collected from the South China Sea. Strains were purified on marine agar 2216E (BD Difco; pH 7.6) at 28°C for 48–72 h and identified via 16S rRNA gene sequencing (primers 27F/1492R) (14). Glycerol stocks (20% vol/vol) were stored at −80°C. The genome data were stored in the GenBank database, and the corresponding GenBank IDs are provided in the supplemental material.

### Genomic sequencing and annotation

Genomic DNA was extracted using the CTAB method (15). DNA libraries were prepared using the NEBNext Ultra II DNA Library Prep Kit (New England Biolabs) according to the manufacturer’s instructions. Whole-genome sequencing was performed on the Illumina NovaSeq 6000 platform (2 × 150 bp paired-end reads).

Raw sequencing reads were quality-checked using FastQC and trimmed with Trimmomatic to remove adapters and low-quality bases. Clean reads were then assembled *de novo* using SPAdes v3.15 (16), and genome annotation was performed with Prokka v1.14.6 (17), with functional assignments based on the KEGG and UniProt databases.

EPS biosynthesis genes (*alg*, *bcs*, *eps*, and *wca*) were identified using antiSMASH v7.0 and manually curated via BLASTp (E-value <1e−5) (18). Phylogenetic analysis was conducted based on the concatenated nucleotide sequences of three housekeeping genes (*rpoB*, *gyrB*, and *recA*). Multiple sequence alignment was performed using MUSCLE in MEGA11 (19). The phylogenetic tree was constructed using the neighbor-joining method with 1,000 bootstrap replicates to evaluate the reliability of the tree topology.

### Fermentation conditions and high-throughput screening

Strains were cultured in the following three media: (i) modified 2216E broth (5 g/L peptone, 1 g/L yeast extract, 0.1 g/L FePO₄, 3% NaCl), (ii) MOPS-buffered minimal medium (pH 7.4), and (iii) a high-carbon medium (20 g/L carbon source). Ten carbon sources (glucose, sucrose, fructose, galactose, glycerol, lactose, maltose, mannitol, xylose, and starch) were tested. Fermentation was conducted in 96-well deep-well plates (1 mL/well) at 28°C and 37°C, 200 rpm, for 72 h. pH was adjusted (5.0–9.0, increments of 1.0) using HCl/NaOH. Growth (OD600) and EPS production were measured every 12 h (20).

### EPS extraction and quantification

EPS was extracted via ethanol precipitation (5). Cultures were centrifuged (10,000 × *g*, 15 min), and the supernatants were mixed with 3× volumes of cold 95% ethanol. Precipitates were dissolved in deionized water and dialyzed (12–14 kDa MWCO) and lyophilized. EPS yields were quantified using the phenol-sulfuric acid method with glucose as a standard (21).

### Statistical analysis and data visualization

All experiments were performed in at least triplicate, and the results are presented as mean ± standard deviation (SD). A two-level factorial design (carbon source × pH × temperature) was analyzed using one-way analysis of variance (ANOVA), followed by Tukey’s Honestly Significant Difference (HSD) post-hoc test to determine significant differences among groups. The significance threshold was set at *P* < 0.05. Prior to ANOVA, assumptions of normality and homogeneity of variance were checked using Shapiro-Wilk and Levene’s tests, respectively.

All statistical analyses were performed in R v4.3.1 (22) using default parameters for post-hoc testing unless otherwise specified. Heatmaps and PCA plots were generated with ggplot2 (23, 24). Genomic pathway diagrams were created using BioRender.com.

## RESULTS

### Hypothetical metabolic pathway for EPS biosynthesis

**Fig 1:**
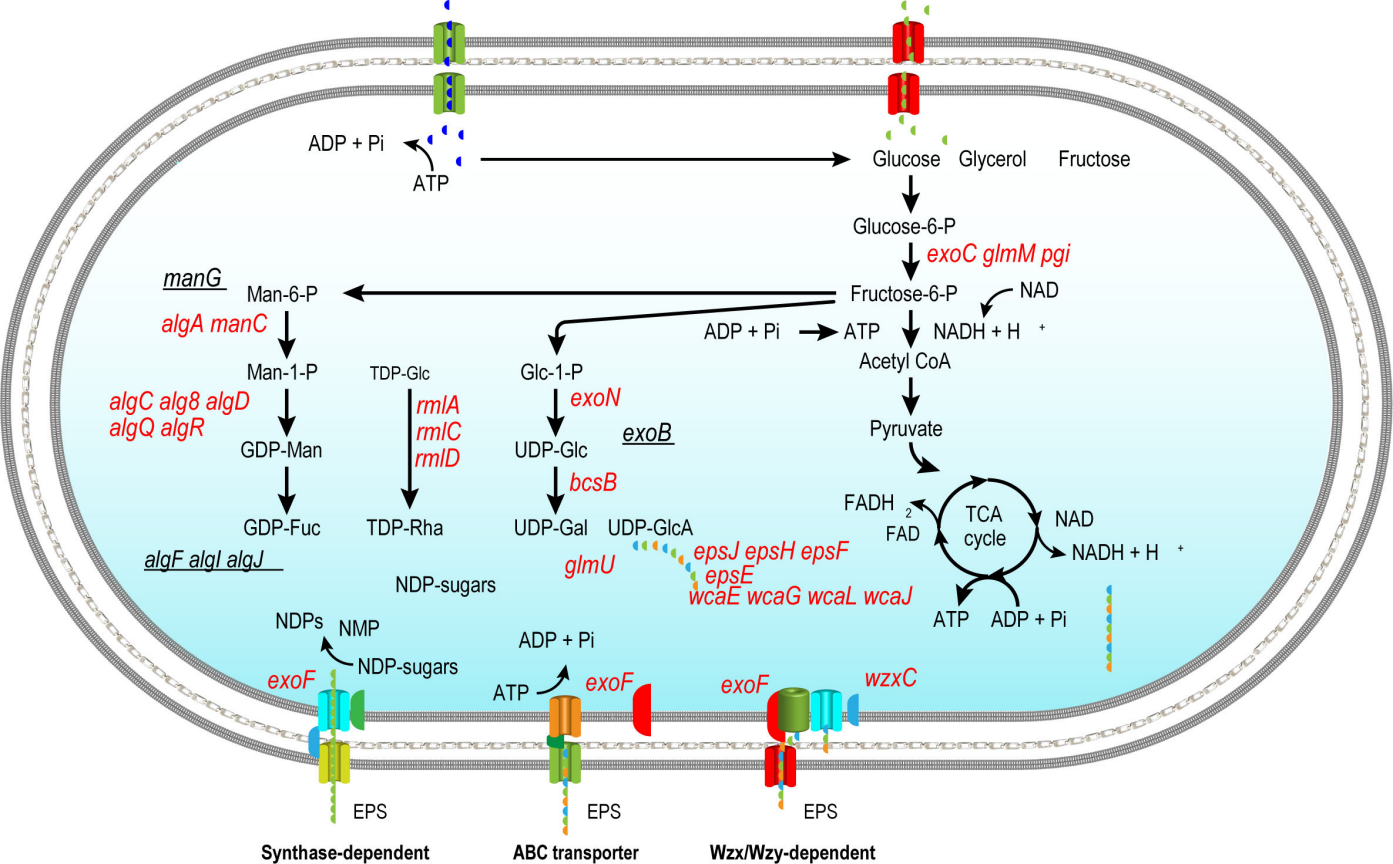
Localization of EPS biosynthesis genes in the metabolic pathway of marine bacteria. Schematic overview showing how key EPS biosynthesis genes are integrated into central carbon metabolism, including glycolysis, the pentose phosphate pathway, and the TCA cycle. Red gene names indicate functionally identified EPS biosynthesis genes with inferred metabolic locations; underlined black genes are also related to EPS biosynthesis, but their precise pathway location is unknown. Main routes for EPS export (synthase-dependent, ABC transporter, and Wzx/Wzy-dependent) are illustrated at the membrane. Abbreviations: ATP: adenosine triphosphate; ADP: adenosine diphosphate; NAD(H): nicotinamide adenine dinucleotide (reduced form); FAD(H₂): flavin adenine dinucleotide (reduced form); GDP: guanosine diphosphate; UDP: uridine diphosphate; TDP: thymidine diphosphate; Man-1-P: mannose-1-phosphate; Glc-1-P: glucose-1-phosphate; GTs: glycosyltransferases; OPX: outer membrane polysaccharide export protein; TCA: tricarboxylic acid cycle.

Based on the genome annotation, a hypothetical metabolic pathway for EPS biosynthesis was constructed (Fig. 1). Key genes involved in the pathway include glycosyltransferases (GTs), polysaccharide co-polymerases (PCPs), and export proteins, which are critical for the synthesis, assembly, and secretion of EPS. The pathway is initiated from central carbon metabolism, where precursors such as glucose-6-phosphate and fructose-6-phosphate are diverted from glycolysis into the pentose phosphate pathway (PPP) to generate nucleotide sugars. These nucleotide sugars serve as activated donors for glycosyltransferase-mediated polymerization.

The annotated gene clusters also suggest the involvement of regulatory proteins, such as two-component systems, which may modulate EPS production in response to environmental cues. This pathway aligns with previously reported metabolic routes in *Bacillus subtilis* (25) and *Pseudomonas fluorescens* (26), providing a foundation for further experimental validation.

### Genomic insights into EPS biosynthetic potential

**Fig 2:**
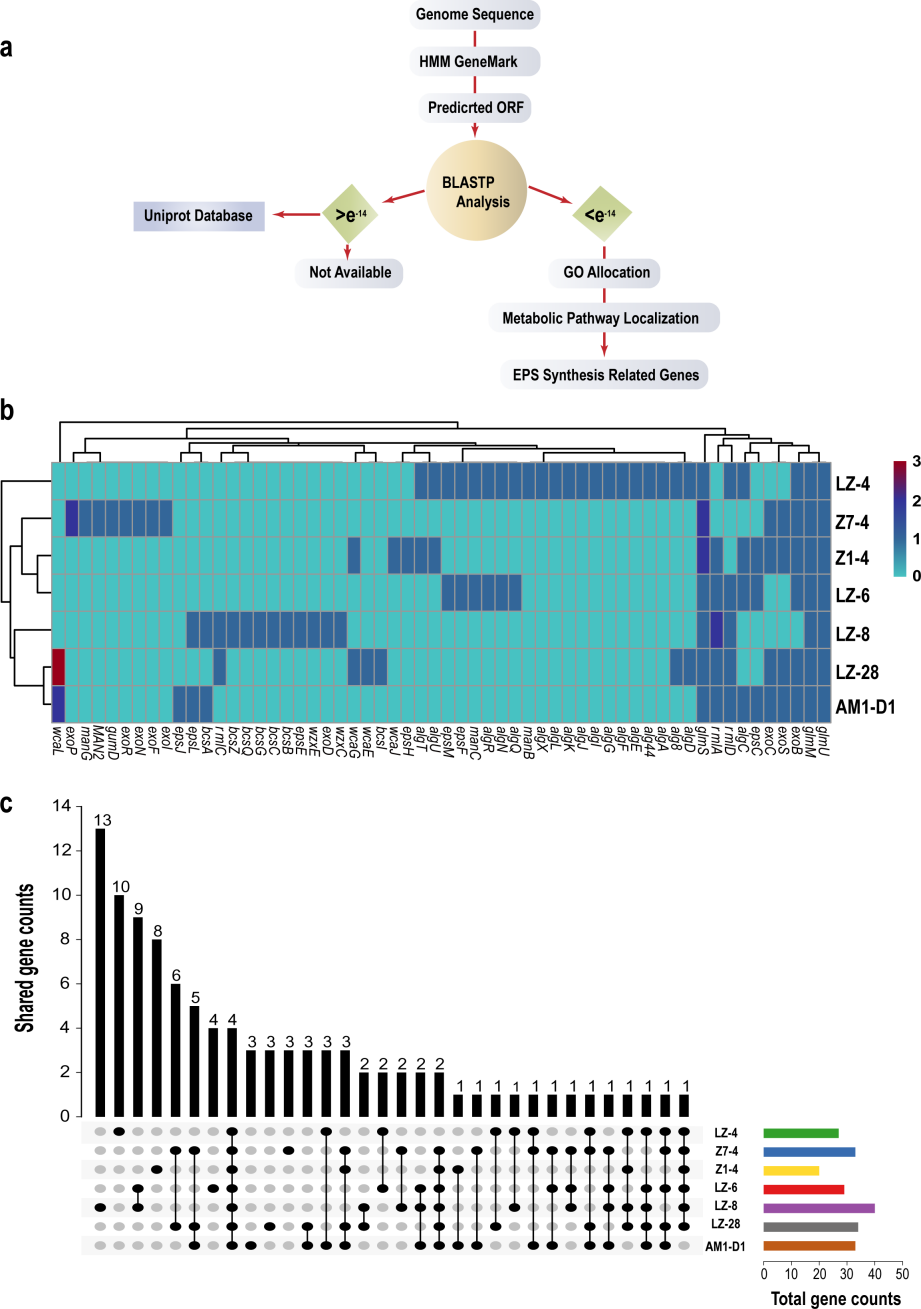
Distribution and comparison of EPS biosynthesis genes in seven marine strains. (**a**) Schematic overview of the genome analysis workflow employed to identify EPS biosynthesis genes. (**b**) Heatmap showing the distribution and abundance of predicted EPS biosynthesis-related genes across seven marine bacterial strains. Strain names are listed on the x-axis, and gene names on the y-axis; color intensity indicates the number of gene copies present in each strain. (**c**) Upset plot illustrating the shared and unique EPS biosynthesis genes among the strains. Horizontal colored bars in the lower left show the total number of EPS biosynthesis genes in each strain. Black dots and connecting lines in the lower right indicate gene intersections among strains, whereas gray slots represent gene absence. Vertical bars in the upper right represent the number of genes in each intersection set, with specific values labeled above the bars. All genes shown were further compared with the UniProt protein database using BLASTP for functional annotation. See Fig. S1 for additional details.

Whole-genome sequencing of the seven novel strains revealed distinct EPS biosynthesis gene clusters (Fig. 2b), based on the genome sequence data in the NCBI following the workflow illustrated in Fig. 2a. The detailed genome accession numbers for all seven marine bacterial strains are provided in Table S1. These genome sequences, previously deposited in GenBank, served as the basis for all subsequent genomic and comparative analyses in this study (Table S1).

*Marinobacter alexandrii* LZ-8 harbored a complete alginate synthesis pathway (*algA/C/D/E*), whereas *Limnobacter alexandrii* LZ-4 exhibited a cellulose synthase operon (*bcsA/B/C*). Strains *Nioella ostreopsis* Z7-4 and *Sulfitobacter alexandrii* AM1-D1 encoded hybrid systems combining *eps* (exopolysaccharide) and *wca* (colanic acid) genes. Phylogenetic analysis based on concatenated housekeeping genes (*rpoB*, *gyrB*, and *recA*) clustered the strains into three clades, correlating with their EPS gene profiles (Fig. 2c). Notably, *Mesorhizobium alexandrii* Z1-4 lacked identifiable EPS clusters despite weak phenol-sulfuric acid detection (8.7 ± 1.2 µg/mL), suggesting either unannotated or novel pathways.

To further evaluate the reliability and coverage of EPS biosynthesis gene annotation, we performed a comparative analysis using both the Gene Ontology (GO) and UniProt databases with “polysaccharide” as the search keyword. The Venn diagram in Fig. S1 illustrates the overlap and unique sets of genes identified by each database. In total, 114 genes were annotated using the GO database and 104 genes using UniProt, with 87 genes shared by both. Notably, 27 genes were uniquely identified by GO and 17 by UniProt, highlighting the complementary nature of these annotation resources (Fig. S1).

### High-throughput fermentation optimization

**Fig 3:**
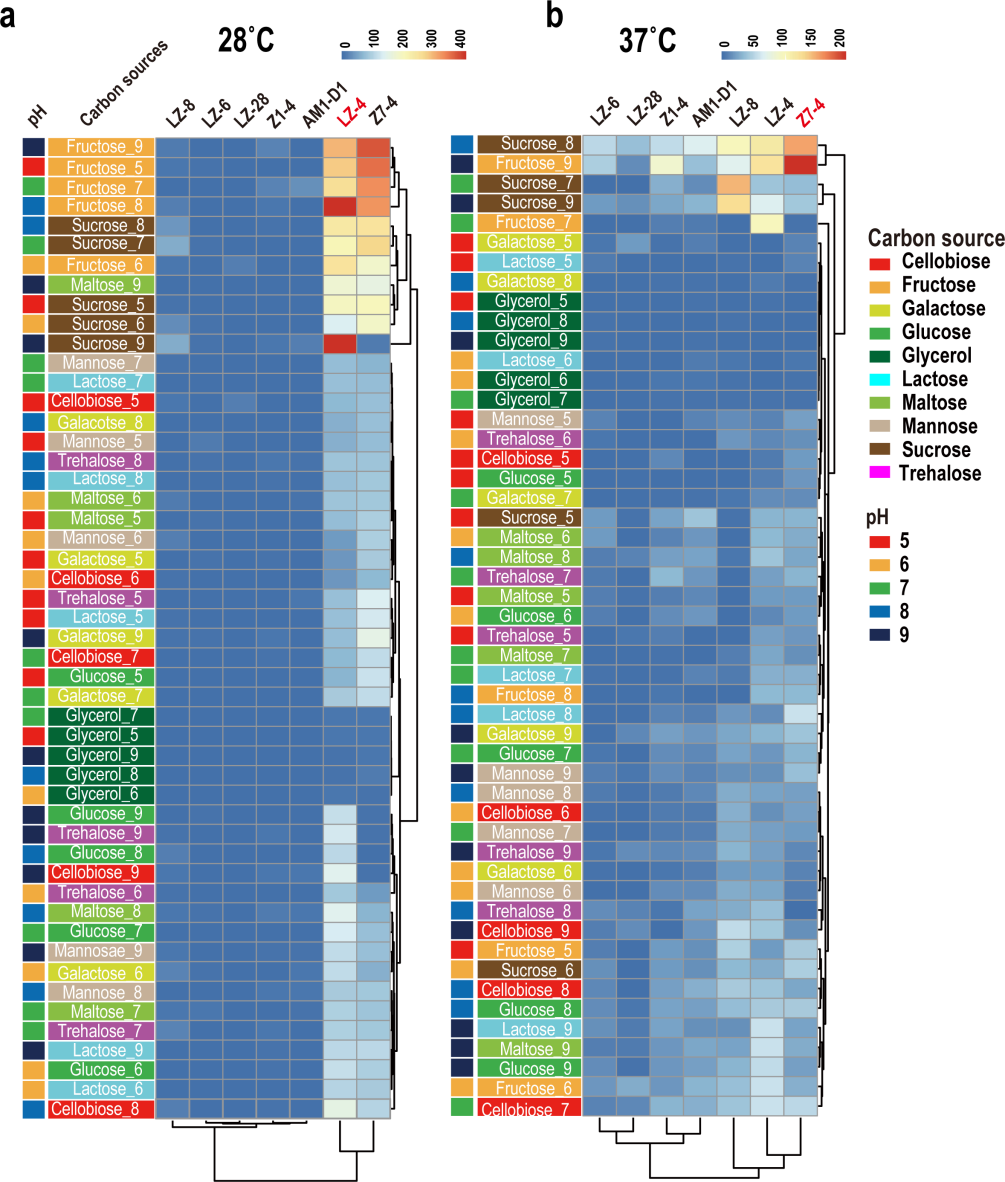
EPS production by seven marine bacterial strains in 50 fermentation media at two temperatures. (**a**) EPS yields (μg/mL) measured after 72 h of fermentation at 28°C; EPS yields (μg/mL) measured after 72 h of fermentation at 37°C. The x-axis represents the seven bacterial strains analyzed, and the y-axis lists the 50 fermentation conditions, each defined by a unique combination of carbon source and pH level. Ten carbon sources are indicated by different background colors (see legend), and five pH levels (5–9) are shown by colored bars on the left. Each cell in the heatmap displays the EPS yield for a given strain and medium, with color intensity ranging from blue (low yield) to red (high yield). Note that the color scales differ between panels (**a**) and (**b**) to reflect the variation in EPS yields at each temperature. Hierarchical clustering was performed using the ‘pheatmap’ package based on Euclidean distance, allowing grouping of strains and conditions with similar EPS production profiles. All EPS yields are reported in μg/mL to ensure unit consistency.

Screening across 50 conditions identified sucrose and fructose as the most effective carbon sources for EPS production (Fig. 3a and b). *L. alexandrii* LZ-4 achieved maximal yields (159.6 ± 12.3 µg/mL) in sucrose-supplemented medium at pH 9 and 28°C. Recent advances in metabolic engineering have enabled *Pseudomonas putida* to serve as a robust platform for the industrial production of exopolysaccharides (EPS), with reported yields typically ranging from 100 to 200 mg/L under optimized conditions (27, 28). In comparison, our best-performing marine isolates achieved EPS yields up to 159.6 µg/mL (approximately 160 mg/L), demonstrating that marine bacteria can match or even exceed the production capacities of established industrial hosts such as *P. putida*. These findings further support the potential of marine strains as sustainable alternatives for industrial EPS bioproduction. Genomic analysis revealed that *L. alexandrii* contains genes involved in cellulose biosynthesis, suggesting that the main EPS produced is cellulose. In contrast, *M. alexandrii* LZ-8 produced 125.2 ± 9.8 µg/mL alginate-like EPS in fructose at pH 7 and 37°C (Fig. 3b). Temperature profoundly influenced strain-specific responses: LZ-4 and Z7-4 showed 40%–50% reduced EPS at 37°C, whereas LZ-8′s yield increased by 32% at elevated temperature. Alkaline conditions (pH 7–9) enhanced EPS secretion across all strains, with pH 9 boosting LZ-4′s yield by 61% compared with pH 5.

### Growth and EPS production dynamics under variable conditions

As a representative strain exhibiting both high EPS yield potential (exceeding 200 µg/mL under optimal conditions) and distinct genomic signatures for heteropolysaccharide biosynthesis (e.g., cellulose-related *bcs* genes), LZ-4 was selected for detailed phenotypic characterization.

**Fig 4:**
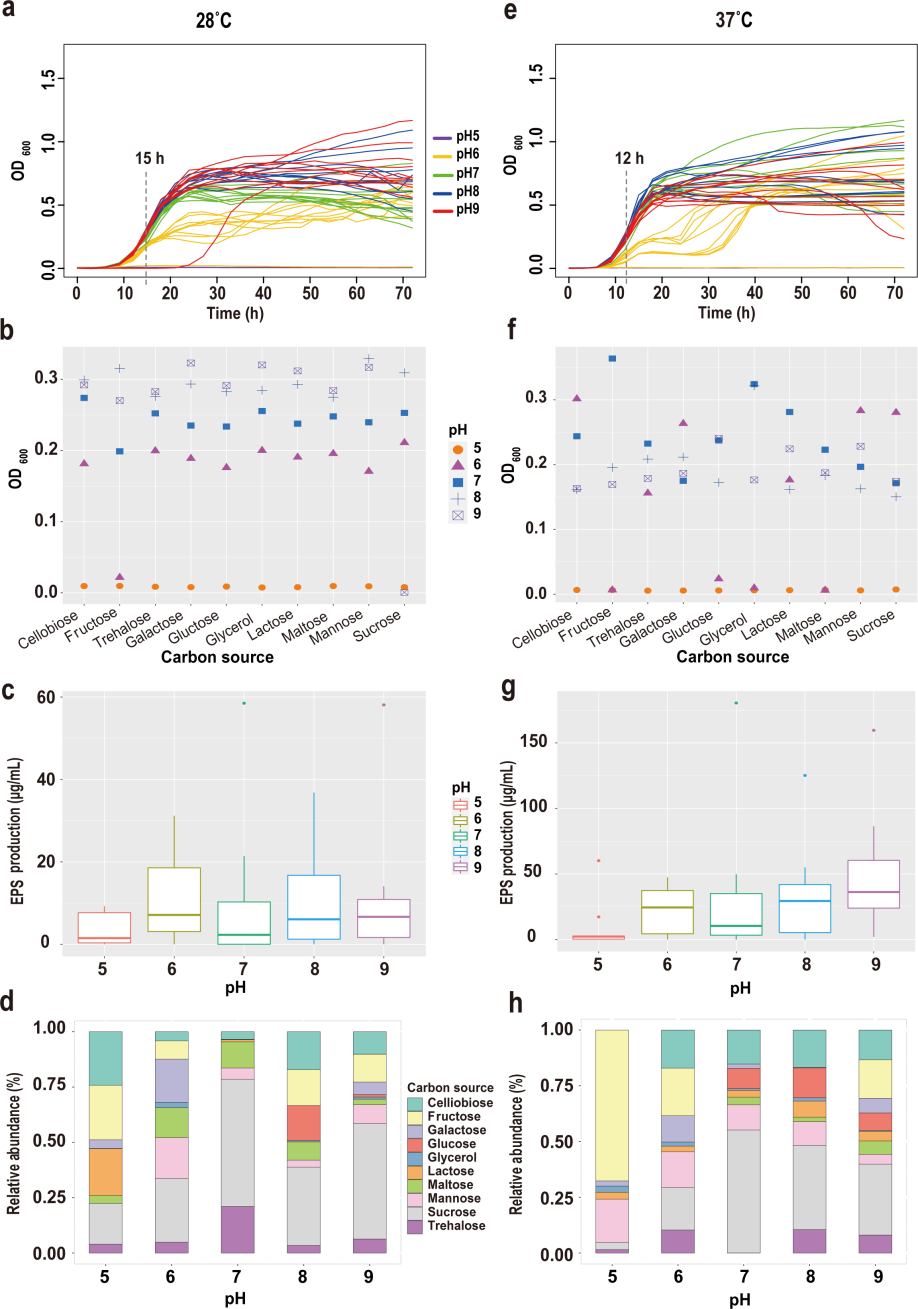
Growth and EPS production of strain LZ-4 under different pH and carbon source conditions at two temperatures. (**a and e**) Growth curves of strain LZ-4 in 50 different media at 28°C (**a**) and 37°C (**e**), with each colored line representing a different pH level (pH 5-9), and each pH including 10 carbon sources. Dashed lines indicate the time points corresponding to the mid-logarithmic phase (15 h for 28°C, 12 h for 37°C). (**b and f**) Optical density (OD₆₀₀) values at the mid-log phase under each of the 50 culture conditions at 28°C (**b**) and 37°C (**f**). Different symbols indicate different pH levels; the x-axis shows the 10 carbon sources. (**c and g**) EPS yields (μg/mL) after 72  h of fermentation under each condition at 28°C (**c**) and 37°C (**g**). Box plots are grouped by pH, each containing 10 carbon source conditions. All yields are reported in μg/mL. (**d and h**) Relative abundance (%) of EPS production contributed by each carbon source at each pH after 72  h at 28°C (**d**) and 37°C (**h**). Stacked bars indicate the proportional contribution of each carbon source to total EPS production at the specified pH. Panels (a–d) show results at 28°C; panels (**e–h**) show results at 37°C. All experiments were performed for 72 h, and the units are consistently presented as indicated.

The growth patterns and EPS yields of LZ-4 across different carbon sources, pH levels, and temperatures are summarized in Fig. 4. The growth performance of all strains in four types of media is shown in the supplemental material; the bacterial strains exhibit the highest growth in 2216E medium compared with other tested media, whereas minimal (1% Glc) medium supports the least growth.

In addition, a summary table of bacterial growth performance for all seven strains across the four tested media is provided in Table S2. This table qualitatively compares the relative growth capacities (“+” to “+++”) observed for each strain under different nutritional and salinity conditions, further illustrating the diversity in environmental adaptability among the isolates (Table S2).

To systematically evaluate the basic physiological characteristics of all seven strains, we compared their growth profiles under a range of fundamental culture conditions. Figure S8 presents the growth curves of all strains in four media types (2216E, 2216E with sea salt, minimal medium with 1% glucose, and MOPS-rich medium with 1.9% NaCl), revealing significant differences in nutrient and salt tolerance. Figure S9 demonstrates the growth dynamics of each strain at two temperatures (28°C and 37°C), highlighting strain-specific thermal adaptation. In addition, Fig. S10 shows the effects of additional carbon supplementation (1% glucose) in 2216E medium at both temperatures, further illustrating the diversity in carbon utilization strategies among the strains. These results establish a comprehensive foundation for interpreting the subsequent optimization of EPS production and phenotypic diversity observed under variable environmental and nutritional conditions (Fig. S8 through S10).

Elevated incubation temperatures significantly altered growth initiation, as cultures at 37°C exhibited a shortened lag phase and earlier entry into the logarithmic growth phase (Fig. 4a and e). Notably, thermal adaptation enabled bacterial proliferation at pH 6 under 37°C, whereas no growth was observed at pH 6 with 28°C incubation. Growth rates demonstrated pH dependency, increasing proportionally across the pH 6–9 range. After 40 h of cultivation, divergent stabilization patterns emerged: at 28°C, cell densities continued rising steadily, particularly under pH 9 with glycerol, galactose, or cellobiose supplementation, whereas 37°C cultures maintained relative stability at pH 8–9 but exhibited fluctuating cell densities at pH 6–7. Carbon source utilization analysis (Fig. 4b and f) revealed glycerol as the optimal substrate for LZ-4 growth at both temperatures, achieving peak growth rates during mid-logarithmic phase. Galactose and lactose supported secondary growth efficiency.

However, when evaluating EPS production, a distinct pattern was observed. However, various carbon sources supported bacterial growth to varying degrees. EPS yields were highly dependent on the type of substrate provided. EPS production displayed pH-dependent enhancement (Fig. 4c and g), increasing progressively with alkalinity despite temperature-induced growth acceleration, showing no stimulatory effect on EPS yields. Carbon source profiling (Fig. 4d and h) identified sucrose and fructose as the primary drivers of EPS biosynthesis, with production levels substantially exceeding those observed with other substrates. The EPS yields obtained with other carbon sources were consistently and significantly lower, highlighting the unique effectiveness of sucrose and fructose for EPS production in LZ-4.

In addition to the detailed analysis of LZ-4 presented in Fig. 4, the growth dynamics and EPS production profiles of the other representative strains—Z7-4, Z1-4, LZ-6, LZ-8, LZ-28, and AM1-D1—under the same carbon sources, pH levels, and temperature conditions are summarized in Fig. S2 through S7. Each supplemental figure provides a comprehensive overview of the growth curves, the mid-log phase OD values, EPS yields, and the relative contribution of each carbon source to EPS production across different pH values and temperature settings. These data reveal strain-specific differences in both growth performance and EPS biosynthetic potential in response to environmental and nutritional variables. Collectively, the results highlight not only common trends (such as the pH dependence of EPS production and the superior performance of certain carbon sources like sucrose and fructose) but also the unique phenotypic characteristics of each strain under diverse culture conditions (Fig. S2 through S7).

Growth curves and EPS production profiles under equivalent experimental conditions demonstrated substantial inter-species variability in both growth kinetics and polymer synthesis capacities. The EPS production of seven marine bacterial strains was significantly enhanced when 1% glucose was added to the 2216E medium, as indicated by the glucose standard curve and comparative analysis. This observation is quantitatively supported by Fig. S11. Panel (a) presents the standard curve for glucose concentration used in EPS quantification, demonstrating high linearity (R² = 0.9998). Panel (b) summarizes the measured EPS yields of all seven strains under both standard (2216E) and glucose-supplemented (2216E + 1% Glc) conditions. The results clearly show that the addition of glucose significantly increased EPS production in most strains, with the greatest enhancement observed in LZ-28 and AM1-D1. These findings confirm that an extra carbon source can substantially boost EPS biosynthetic capacity in marine bacteria (Fig. S11).

### Correlation between bacterial growth and EPS production

**Fig 5:**
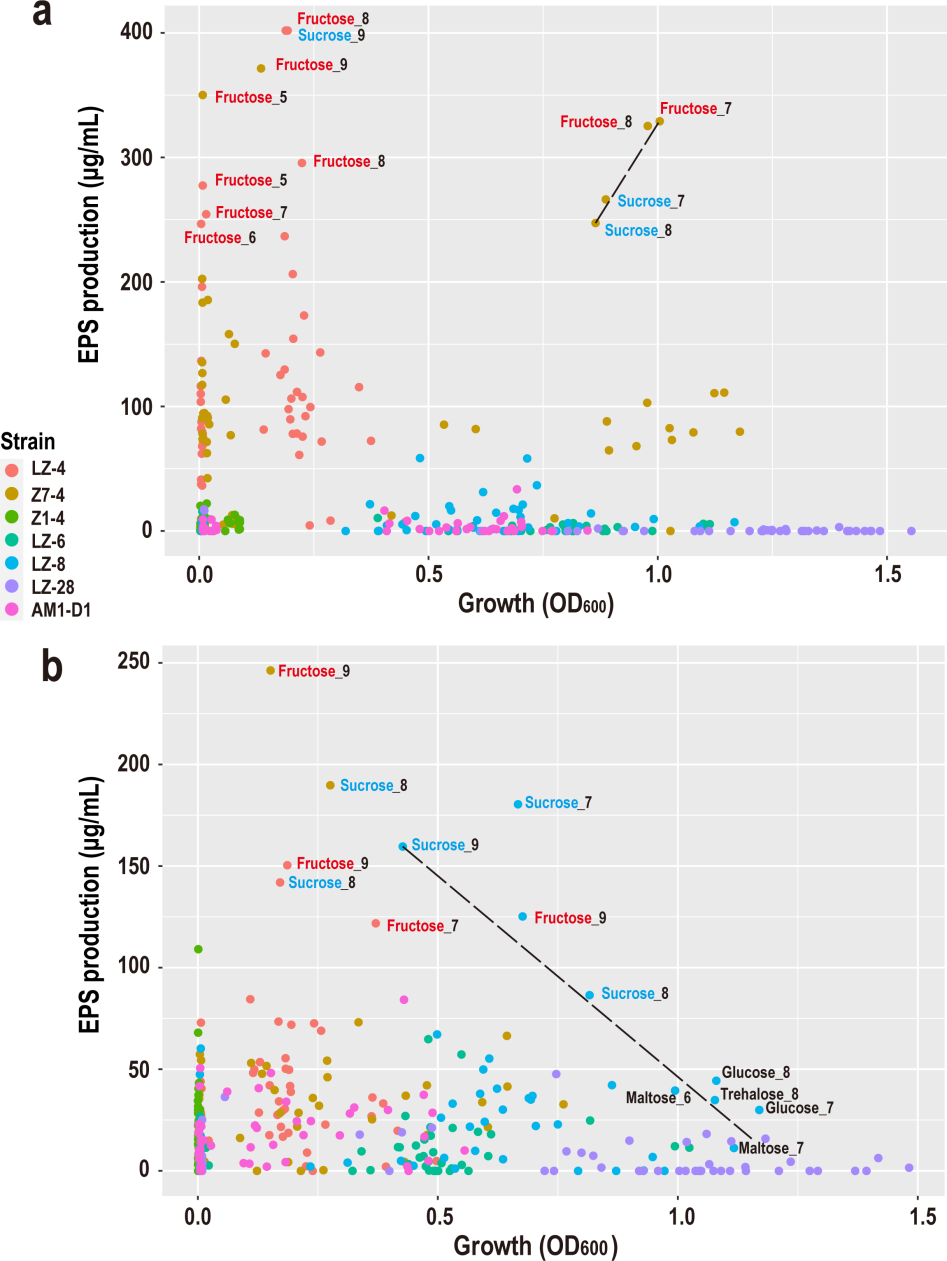
Correlation between bacterial growth (OD 600) and EPS production by seven marine bacterial strains under 50 fermentation conditions at two temperatures. (**a**) Scatter plot of EPS yield versus bacterial growth (OD600) for all strains after 72 h of fermentation at 28°C. (**b**) Scatter plot of EPS yield versus bacterial growth (OD600) at 37°C. Each point represents a unique combination of strain, carbon source, and pH condition (a total of 50 conditions per strain). The x-axis shows bacterial growth measured as OD600, whereas the y-axis shows EPS production in units of μg/mL. Different colors indicate different bacterial strains, as shown in the legend. Data points with the highest EPS yields are labeled with their corresponding carbon source and pH value (e.g., "Fructose_8" indicates fructose as the carbon source at pH 8). A total of seven strains were tested, and each was cultivated in 50 fermentation media at both 28°C and 37°C. All measurements were taken after 72 h of incubation.

Cluster analysis of OD values and EPS production in seven marine bacterial strains revealed distinct growth characteristics, with densely clustered points indicating similar phenotypic traits. As shown in Fig. 5, strains LZ-4 and Z7-4 exhibited the highest EPS yields among the tested strains. Notably, EPS production in these two strains showed no significant correlation with bacterial growth. For Z7-4, EPS levels increased with rising OD values within the range of 0.75–1.0 (Fig. 5a). However, LZ-4 displayed substantial fluctuations in EPS yield at OD600 equal to 0.25, influenced by pH variations and carbon source availability. Intriguingly, both Z7-4 and LZ-4 maintained relatively high EPS production under fructose-supplemented conditions. In contrast, EPS yields in Z1-4, LZ-6, LZ-8, LZ-28, and AM1-D1 remained growth-independent, showing minimal variation across OD values. Temperature elevation significantly enhanced EPS synthesis in LZ-8, particularly with sucrose or fructose as carbon sources, achieving yields exceeding 125 µg/mL. Paradoxically, under specific growth conditions (denoted by black dashed lines in Fig. 5b), LZ-8 exhibited an inverse relationship between growth rate and EPS production, irrespective of the carbon source.

The optimal fermentation conditions for maximal EPS biosynthesis varied substantially among strains (Table 1). Although Z7-4 exhibited a preference for neutral pH in terms of growth, all other strains demonstrated alkaliphilic tendencies. Notably, the ideal parameters for EPS synthesis diverged markedly from those supporting optimal growth across all strains, suggesting that peak EPS production does not coincide with maximal biomass accumulation. Notably, the optimal conditions for EPS synthesis consistently differed from those supporting maximal growth across all strains, indicating that peak EPS production is uncoupled from biomass accumulation. Carbon source preference, temperature, and pH requirements were strain-specific, underscoring the metabolic diversity of these marine isolates.

**TABLE 1:** Summary of optimum fermentation conditions and EPS biosynthesis conditions of seven marine bacteria

Strain	Optimal growth condition			Optimal EPS production condition		
	Temperature (°C)	Carbon source	pH level	Temperature (°C)	Carbon source	pH level
LZ-4^*a*^	37	Glycerol	9	28	Fructose	8
Z7-4^*b*^	28	Cellulose	7	28	Fructose	9
Z1-4	28	Galactose	8	37	Fructose	9
LZ-6	37	Maltose	9	37	Sucrose	8
LZ-8	37	Glucose	8	37	Sucrose	7
LZ-28	37	Cellulose	8	37	Sucrose	8
AM1-D1	28	Glucose	8	37	Sucrose	8

^*a*^ Highest EPS production.

^*b*^ Relatively high EPS production.

## DISCUSSION

### Mechanistic insights into EPS biosynthesis

This study elucidates the potential mechanisms underlying EPS biosynthesis in seven novel marine bacterial strains through genomic analysis. The identification of glycosyltransferases and related enzymes highlights their essential role in catalyzing the polymerization of nucleotide sugars into high-molecular-weight polysaccharides. These findings align with established pathways observed in model organisms such as *Lactobacillus plantarum* (29). Furthermore, regulatory genes, including two-component systems, suggest that EPS production may be modulated by environmental cues, as seen in *Sinorhizobium meliloti* (30).

Although direct transcriptomic and metabolomic data are absent, the annotated genes provide indirect but robust support for the proposed pathway. For instance, previous transcriptomic studies in *Pseudomonas aeruginosa* revealed upregulation of EPS-related genes under nutrient-limited conditions (31, 32), whereas metabolomic profiling of *Bacillus subtilis* demonstrated the accumulation of nucleotide sugars as EPS precursors (33). Future integration of multi-omics approaches could validate these mechanisms and uncover additional regulatory elements, paving the way for more precise engineering of EPS biosynthesis.

### Bridging marine ecology and industrial biotechnology

The genomic and phenotypic divergence among the seven strains underscores the influence of marine phytoplankton symbiosis in driving functional specialization for EPS biosynthesis. For instance, *Marinobacter alexandrii* LZ-8′s alginate pathway aligns with its ecological role in biofilm formation on dinoflagellate surfaces—a trait likely co-opted from its host’s extracellular matrix stabilization requirements (34). Similarly, *Limnobacter alexandrii* LZ-4′s cellulose production reflects adaptation to the polysaccharide-rich phycosphere, where structural EPS aids in nutrient retention (35). These findings highlight the potential of “eco-engineering” as a strategy to harness niche-specific microbial traits for industrial applications (36).

### Potential industrial applications and feasibility

Although being conducted on a small scale using 96-well plate fermentation, the study demonstrates significant industrial potential. Theoretical extrapolation to industrial-scale fermenters (e.g., 10,000 L) suggests the production levels of approximately 2–5 g/L, comparable with or exceeding reported yields of industrial strains such as *Bacillus subtilis* and *Lactiplantibacillus plantarum* (37, 38).

A key advantage of the proposed strains lies in their ability to utilize inexpensive carbon sources, such as sucrose and fructose, which are widely available as agricultural by-products. This could substantially reduce production costs compared with conventional EPS systems relying on glucose. Furthermore, integrating EPS production with waste valorization processes, such as utilizing food or agricultural waste streams, offers further pathways to enhance cost efficiency and sustainability.

However, scaling up EPS production presents challenges, including oxygen transfer limitations, pH control, and substrate inhibition. Bioreactor designs with enhanced aeration and mixing efficiency, such as fed-batch or continuous systems, should be evaluated. Additionally, optimizing upstream processes, including inoculum preparation and media composition, will be critical for consistent performance at scale.

### Advantages and challenges of high-throughput screening

High-throughput screening (HTS) played a critical role in this study, significantly accelerating strain development and fermentation optimization. Compared with traditional one-factor-at-a-time approaches, this HTS strategy reduced optimization time by 70%, achieving strain-specific condition refinement within 10 days (39). This efficiency is comparable to or exceeds that reported in recent studies, such as Freitas et al. (5, 9,), where HTS enabled rapid identification of high-yielding strains and optimal conditions for EPS production within 2 weeks. The parallel testing capability of our HTS platform facilitated the simultaneous evaluation of multiple fermentation variables, aligning well with industrial demands for high-throughput, cost-effective bioprocesses (40). Nevertheless, similar to previous reports, our HTS approach is limited by the scale of microplate-based assays, which may not fully recapitulate the shear stress and oxygen transfer dynamics of larger bioreactors (40, 41). Therefore, as highlighted in prior studies, results from HTS platforms should be validated at larger scales to ensure industrial relevance and scalability.

### Potential structural and functional properties of EPS

Although detailed chemical characterization of the EPS was not performed, insights from similar studies provide a basis for hypothesizing their structural and functional properties. For example, EPS from *Bacillus subtilis* and *Lactiplantibacillus plantarum* typically exhibit molecular weights ranging from 10⁵ to 10⁶ Da and are composed of glucose, galactose, and mannose as primary monosaccharide units (42, 43). These polysaccharides often feature branched structures with β-1,3 and β-1,6 linkages, which contribute to enhancing bioactivities, such as antioxidant and immunomodulatory properties (44). In comparison, previous studies on marine-derived EPS, such as those from Pseudoalteromonas and Vibrio species, have reported higher sulfate content and unique monosaccharide compositions, contributing to increased metal chelation and emulsification capacities (1, 10). Our preliminary results suggest that the EPS produced by LZ-4 may share similar structural features with terrestrial Bacillus EPS, but further analysis is required to confirm potential marine-specific adaptations, such as higher salt tolerance or unique functional groups. Thus, our findings contribute to the growing body of knowledge on both the diversity and potential applications of microbial EPS and highlight the need for comprehensive structural and functional analyses in future work.

Future studies employing advanced techniques such as gel permeation chromatography (GPC), nuclear magnetic resonance (NMR), and Fourier-transform infrared spectroscopy (FTIR) will provide definitive insights into the chemical nature and potential applications of these EPS.

### Genome-guided optimization and limitations

Although genomic predictions successfully identified high-yield strains (e.g., LZ-8 and LZ-4), the poor correlation observed in *Mesorhizobium alexandrii* Z1-4 highlights critical gaps in current annotation tools. Both AntiSMASH and BLASTp failed to detect non-canonical EPS clusters, suggesting the existence of novel biosynthetic pathways in understudied marine taxa (45). This underscores the necessity of hybrid approaches combining deep learning-based gene prediction (e.g., DeepEC [46]) with metabolomic profiling to uncover "cryptic" EPS pathways.

### Toward sustainable marine bioeconomies

This study directly contributes to the United Nations Sustainable Development Goal (SDG) 14 ("Life Below Water") by valorizing marine microbial resources without habitat disruption. With global EPS demand projected to reach 2.1 million tons by 2030 (47), transitioning from terrestrial feedstocks (e.g., corn for xanthan gum) to marine bacteria could reduce agricultural water consumption by 40%–60% per ton produced (48).

However, scaling marine EPS production requires addressing bottlenecks in biomass pretreatment and salt-tolerant fermentation engineering. Future efforts should focus on process optimization to ensure commercial viability and environmental sustainability (49).

### Limitations and future directions

The following two key limitations persist in this study: (i) the lack of *in situ* transcriptomic data to correlate gene expression with EPS yield dynamics, and (ii) unresolved shear stress effects on EPS rheology during scaled fermentation.

Future work should integrate multi-omics (RNA-seq, proteomics) with computational fluid dynamics modeling to better predict industrial performance (50). Additionally, detailed functional assays and pilot-scale production trials are necessary to validate the potential applications of marine-derived EPS. These include antioxidant properties for food preservation, biocompatibility for tissue engineering, and biodegradability for environmental applications.

### Conclusion

This study provides a comprehensive genomic-phenomic analysis of seven novel marine bacterial strains, highlighting their remarkable EPS biosynthesis capabilities shaped by ecological specialization within algal phycospheres. Key findings include the discovery of strain-specific pathways, such as the complete alginate synthesis operon in *Marinobacter alexandrii* LZ-8 and the cellulose synthase system in *Limnobacter alexandrii* LZ-4, which demonstrate how host-microbe coevolution adapts biopolymer functionality to ecological roles like biofilm stabilization and nutrient retention. Another key finding is the development of a high-throughput fermentation platform, which significantly accelerated EPS yield optimization and reduced traditional timelines by 70%.

These findings address a critical bottleneck in microbial bioprospecting and enhance the feasibility of scaling marine-derived biopolymer production. Moreover, the study emphasizes the potential of these EPS for industrial applications, including bioplastics, medical materials, and environmental remediation while contributing to global circular bioeconomy goals.

Future research should prioritize integrating AI-driven pathway prediction, CRISPR-based activation of silent gene clusters, and large-scale phenotypic characterization. These approaches will unlock the full potential of marine microbial resources, enabling the discovery of novel biopolymers and advancing next-generation material science.

### Highlights

- Marine bacterial strains were screened for high-yield exopolysaccharide (EPS) production.
- Genomic analysis identified key EPS biosynthesis genes and metabolic pathways.
- High-throughput fermentation optimized EPS yields using sucrose and alkaline pH.
- Strain-specific conditions (sucrose as the carbon source, pH 9, and 28°C) enhanced EPS production up to 159.6 μg/mL.
- The study provides a scalable framework for sustainable industrial EPS production.

## Data Availability

Genomic data have been deposited in GenBank, with accession numbers listed in the supplemental material.

## SUPPLEMENTAL MATERIAL

Supplemental material aem.00837-25-s0001.docx Tables S1 and S2; Fig. S1 to S11.

ASM does not own the copyrights to Supplemental Material that may be linked to, or accessed through, an article. The authors have granted ASM a non-exclusive, world-wide license to publish the Supplemental Material files. Please contact the corresponding author directly for reuse.
