# Supplementary material for: High-throughput and genome-guided optimization of exopolysaccharide production in marine bacteria for sustainable biotechnology
Source: Appl Environ Microbiol. 2025 Oct 21;91(11):e00837-25. doi: 10.1128/aem.00837-25 (PMC12628687; doi:10.1128/aem.00837-25)
Supplement: Supplemental material — Tables S1 and S2; Fig. S1 to S11. [file aem.00837-25-s0001.docx]

**Supporting Information**

**High-Throughput and Genome-Guided Optimization of Exopolysaccharide Production in Marine Bacteria for Sustainable Biotechnology**

Yajun Wei^2#^, Fang Zhou^3#^, ZhangJian Feng^2^, Min Qi^2^, Rong Xiang^4^, Hongbo Yi^2^, Qiao Yang^5*^, Xi Yang^1*^

1. Fuyao University of Science and Technology, Fuzhou 350100, Fujian, China
2. State Key Laboratory of Swine and Poultry Breeding Industry, Key Laboratory of Animal Nutrition and Feed Science in South China, Ministry of Agriculture and Rural Affairs, Guangdong Provincial Key Laboratory of Animal Breeding and Nutrition, Institute of Animal Science, Guangdong Academy of Agricultural Sciences, Guangzhou 510642, China
3. Sericultural and Agri-Food Research Institute, Guangdong Academy of Agricultural Sciences, Key Laboratory of Functional Foods, Ministry of Agriculture and Rural Affairs, Guangdong Key Laboratory of Agricultural Products Processing, Guangzhou 510610, China，
4. Institute of Animal Health, Guangdong Academy of Agricultural Sciences, Guangzhou 510640, China
5. ABI Group, Phycosphere Microbiology Laboratory, Zhejiang Ocean University, Zhoushan 316022, China

#Yajun Wei and Fang Zhou contributed equally to this work as first authors.

* XiYang and Qiao Yang contributed to this work as corresponding authors, Fuyao University of Science and Technology, Fuzhou 350100, Fujian, China. E-mail address: [xishelleyyang@126.com](mailto:xishelleyyang@126.com)

This supplementary materials contain 14-page document, including 2 tables, 11 figures and this cover page.

**Table S1 Genome sequence ID of 7 marine bacteria**

| **Strain** | **Genome ID** |
| --- | --- |
| *Limnobacter alexandrii* LZ-4 | SWKN01 |
| *Nioella ostreopsis* Z7-4 | RCIO01 |
| *Mesorhizobium alexandrii* Z1-4 | RCIO01 |
| *Marinobacter shengliensis subsp. alexandrii* LZ-6 | SWKL01 |
| *Marinobacter alexandrii* LZ-8 | SWKM01 |
| *Memelialla alexandrii* LZ-28 | JAANYX01 |
| *Sulfitobacter alexandrii* AM1-D1 | CP018076 |

Note: The genome sequences of these seven marine bacteria were previously reported and were not sequenced in this study. Among them, strain AM1-D1 has a complete genome sequence, whereas the remaining six strains have draft genome assemblies. All genomic data are publicly available in the GenBank database.

**Tab S2 Summary of bacterial growth in four kinds of mediums**

| **Strain** | **2216E** | **2216E (sea salt)** | **Minimal (1% Glc)** | **MOPS-rich (1.9% NaCl)** |
| --- | --- | --- | --- | --- |
| LZ-4 | + | +++ | - | ++ |
| Z7-4 | +++ | ++ | - | - |
| Z1-4 | +++ | - | - | - |
| LZ-6 | + | ++ | - | +++ |
| LZ-7 | +++ | ++ | - | + |
| LZ-28 | ++ | +++ | - | - |
| AM1-D1 | ++ | + | - | +++ |

Note: "+" denotes enhanced bacterial growth performance, while "-" indicates no observable bacterial growth.

**
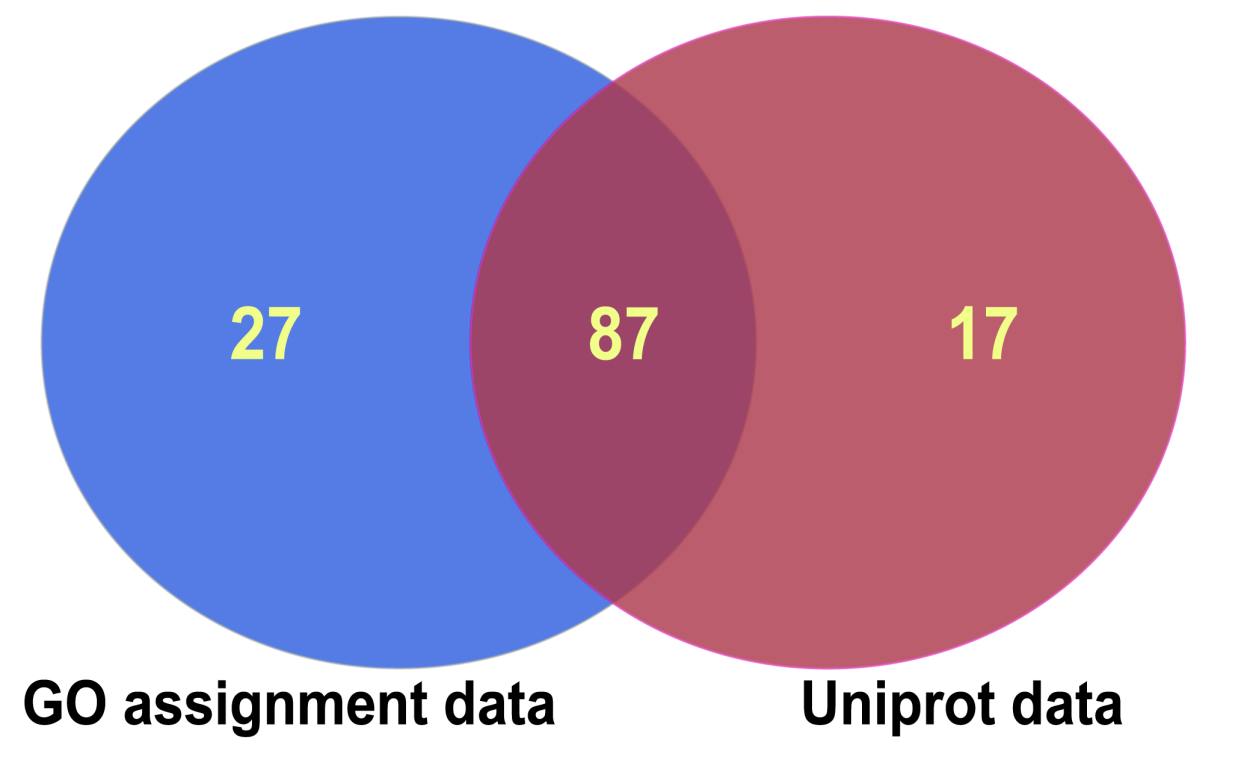
Figure S1. Venn diagram of comparison of two search results for predicting EPS biosynthesis related genes.** The Blue circle represents the genes retrieved from a Gene Ontology (GO) annotation search using the key word "polysaccharide". The Red circle indicates the genes identified through a Uniprot database search for EPS biosynthesis genes using the same keyword. The overlapping purple area represents the intersection between these two databases. Numbers inside the circles indicate the gene counts. All gene matches have an evalue <e-14.

**
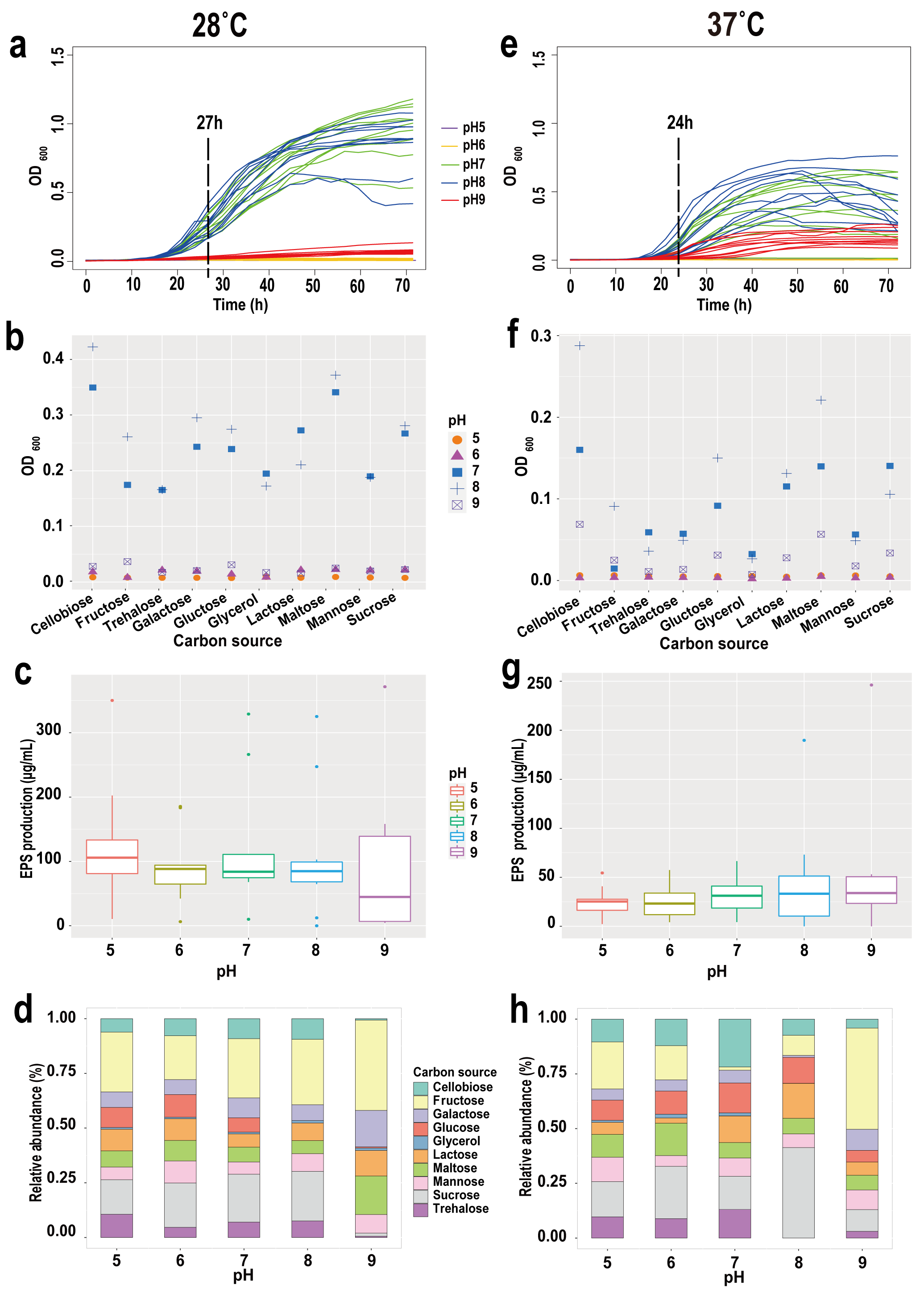
Figure S2. Bacterial growth OD value and EPS production of Z7-4 cultured under different conditions for 72h.** (a, e) Bacterial growth curves under different PH levels, each comprising ten carbon source conditions. Dashed lines indicate the mid-log phase time points; (b, f) OD values at the mid-log phase time points across 50 distinct culture conditions; (c, g) EPS production (μg/mL) after 72 hours of fermentation. Different colored box plots correspond to different pH levels, with each pH group containing ten carbon source conditions; (d, h) Relative EPS yield per carbon source at each pH level after 72 hours of fermentation. Panels (a), (b), (c), and (d) present data measured at 28 °C, while panels (e), (f), (g), and (h) present data measured at 37 °C.

**
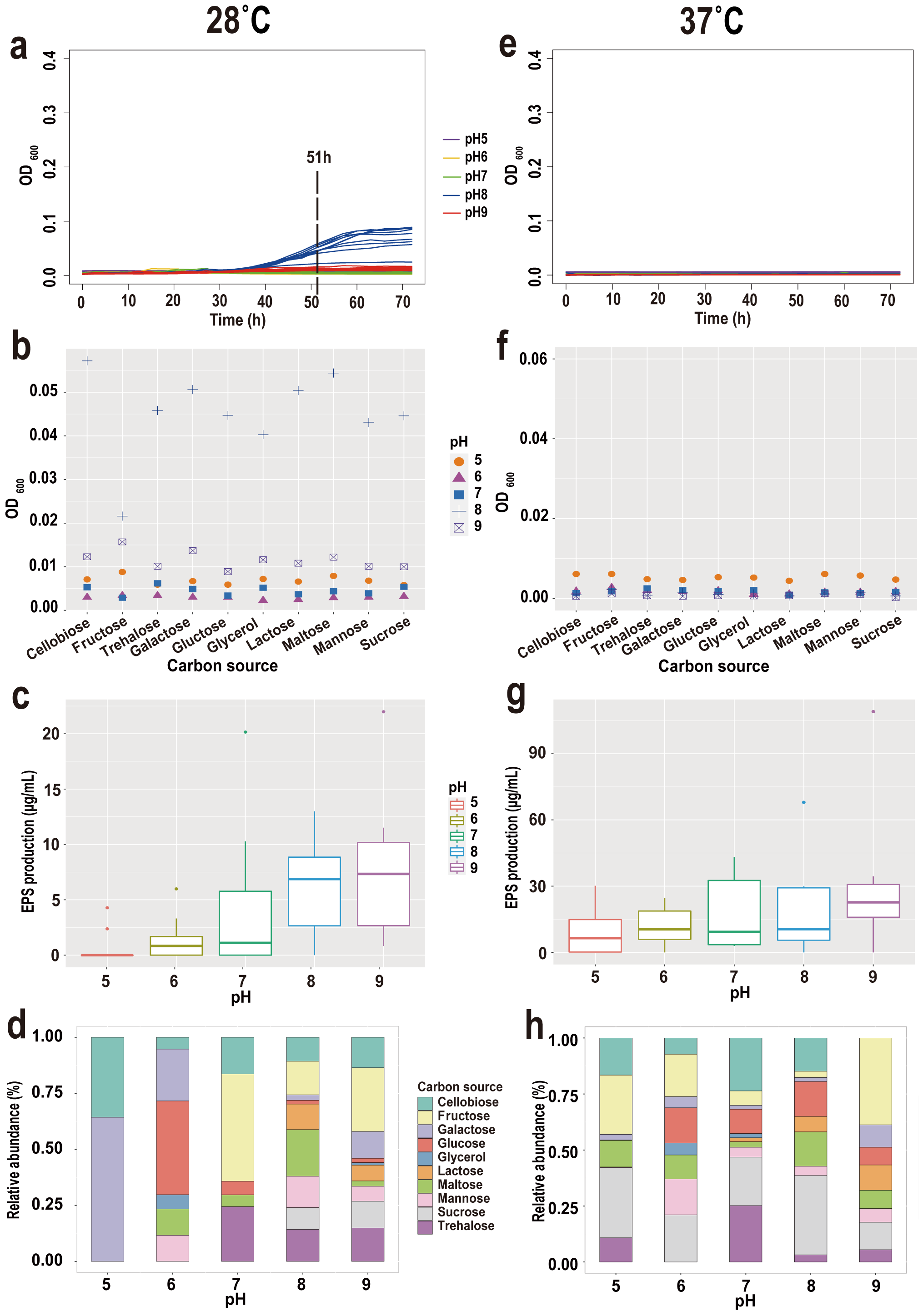
Figure S3. Bacterial growth OD value and EPS production of Z1-4 cultured under different conditions for 72h.** (a, e) Bacterial growth curves under different PH levels, each comprising ten carbon source conditions. Dashed lines indicate the mid-log phase time points; (b, f) OD values at the mid-log phase time points across 50 distinct culture conditions; (c, g) EPS production (μg/mL) after 72 hours of fermentation. Different colored box plots correspond to different pH levels, with each pH group containing ten carbon source conditions; (d, h) Relative EPS yield per carbon source at each pH level after 72 hours of fermentation. Panels (a), (b), (c), and (d) present data measured at 28 °C, while panels (e), (f), (g), and (h) present data measured at 37 °C.

**
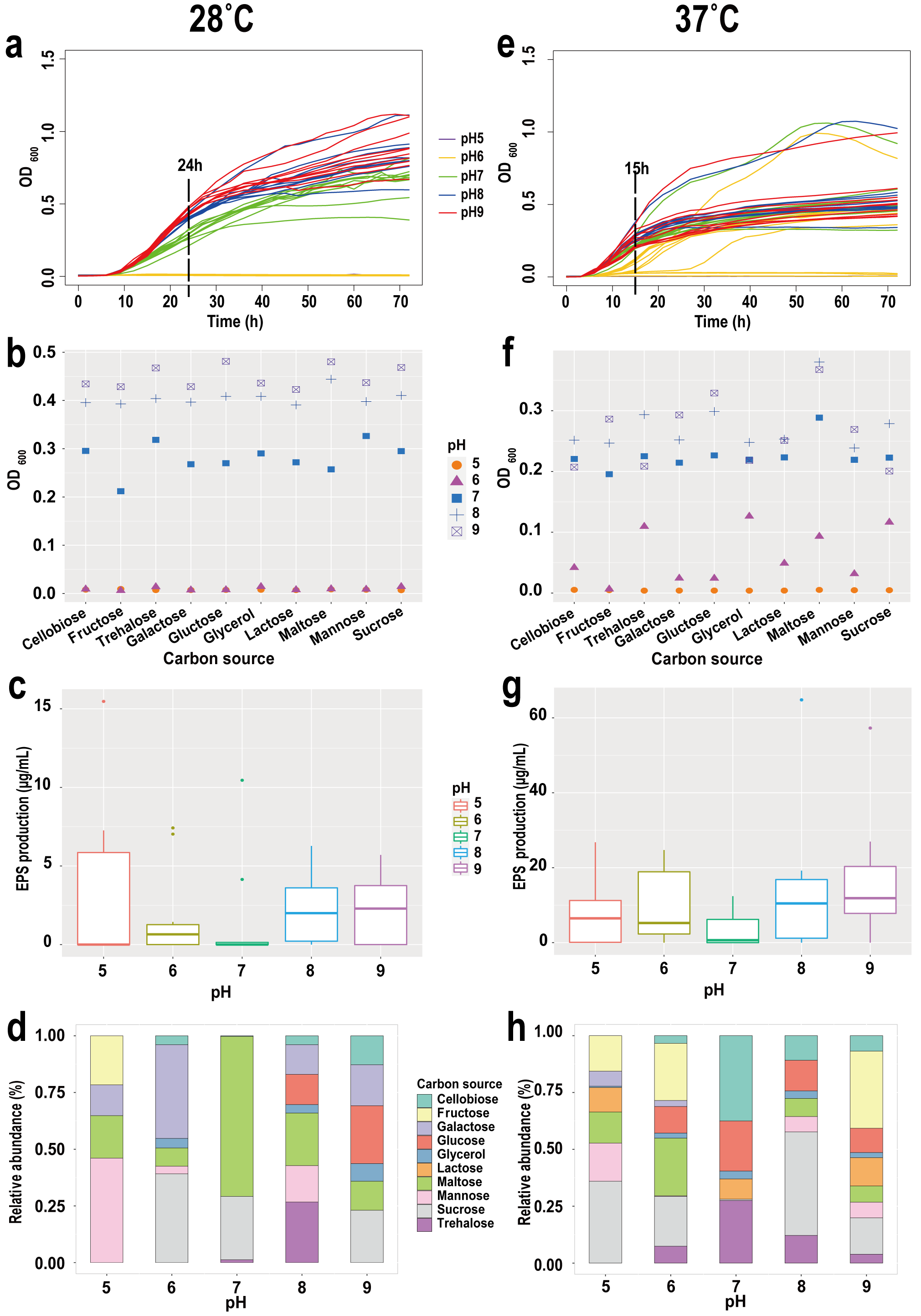
Figure S4. Bacterial growth OD value and EPS production of LZ-6 culturedunder different conditions for 72h.** (a, e) Bacterial growth curves under different PH levels, each comprising ten carbon source conditions. Dashed lines indicate the mid-log phase time points; (b, f) OD values at the mid-log phase time points across 50 distinct culture conditions; (c, g) EPS production (μg/mL) after 72 hours of fermentation. Different colored box plots correspond to different pH levels, with each pH group containing ten carbon source conditions; (d, h) Relative EPS yield per carbon source at each pH level after 72 hours of fermentation. Panels (a), (b), (c), and (d) present data measured at 28 °C, while panels (e), (f), (g), and (h) present data measured at 37 °C.

**
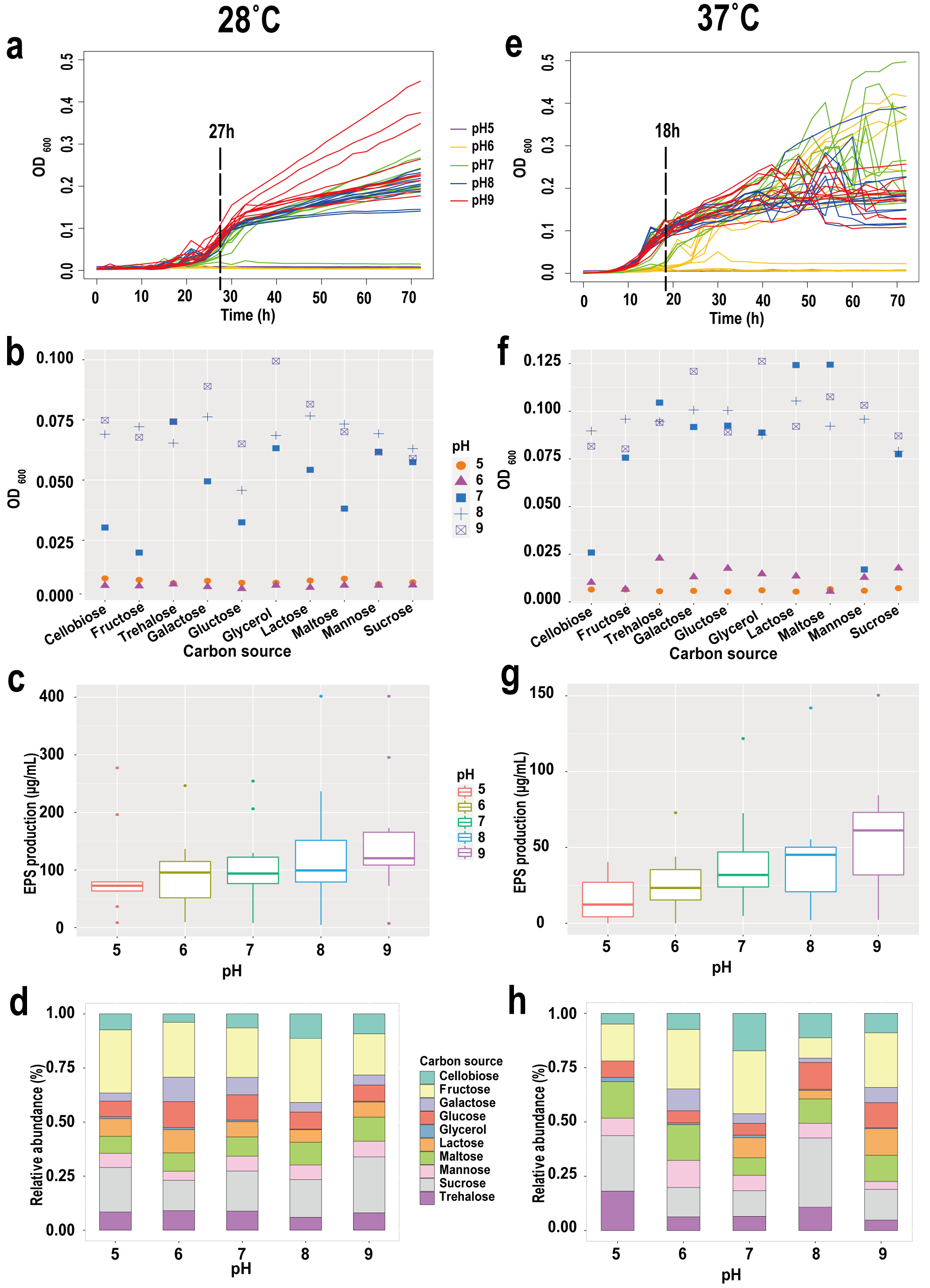
Figure S5. Bacterial growth OD value and EPS production of LZ-8 cultured under different conditions for 72h.** (a, e) Bacterial growth curves under different PH levels, each comprising ten carbon source conditions. Dashed lines indicate the mid-log phase time points; (b, f) OD values at the mid-log phase time points across 50 distinct culture conditions; (c, g) EPS production (μg/mL) after 72 hours of fermentation. Different colored box plots correspond to different pH levels, with each pH group containing ten carbon source conditions; (d, h) Relative EPS yield per carbon source at each pH level after 72 hours of fermentation. Panels (a), (b), (c), and (d) present data measured at 28 °C, while panels (e), (f), (g), and (h) present data measured at 37 °C.

**
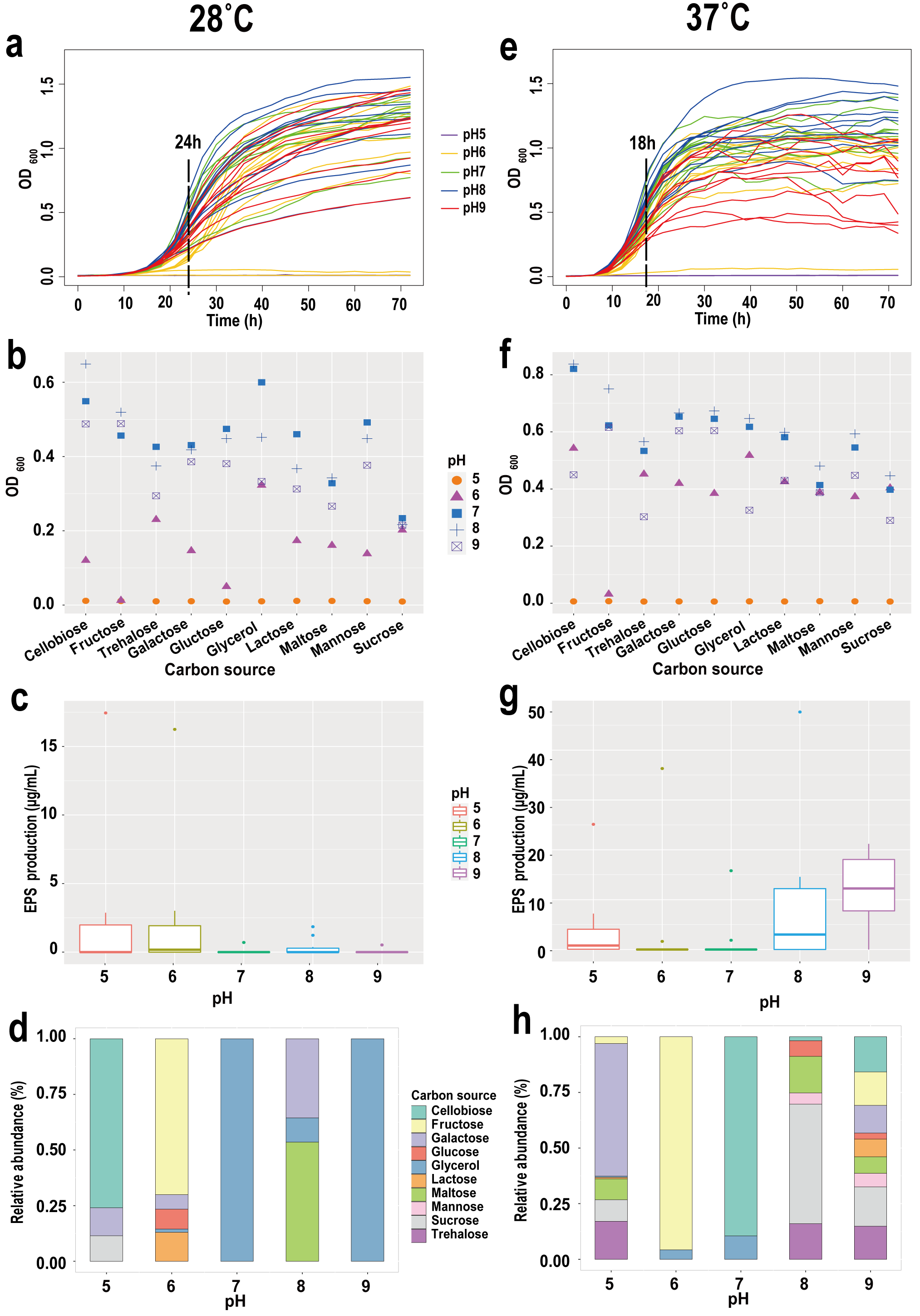
Figure S6. Bacterial growth OD value and EPS production of LZ-28 cultured under different conditions for 72h.** (a, e) Bacterial growth curves under different PH levels, each comprising ten carbon source conditions. Dashed lines indicate the mid-log phase time points; (b, f) OD values at the mid-log phase time points across 50 distinct culture conditions; (c, g) EPS production (μg/mL) after 72 hours of fermentation. Different colored box plots correspond to different pH levels, with each pH group containing ten carbon source conditions; (d, h) Relative EPS yield per carbon source at each pH level after 72 hours of fermentation. Panels (a), (b), (c), and (d) present data measured at 28 °C, while panels (e), (f), (g), and (h) present data measured at 37 °C.


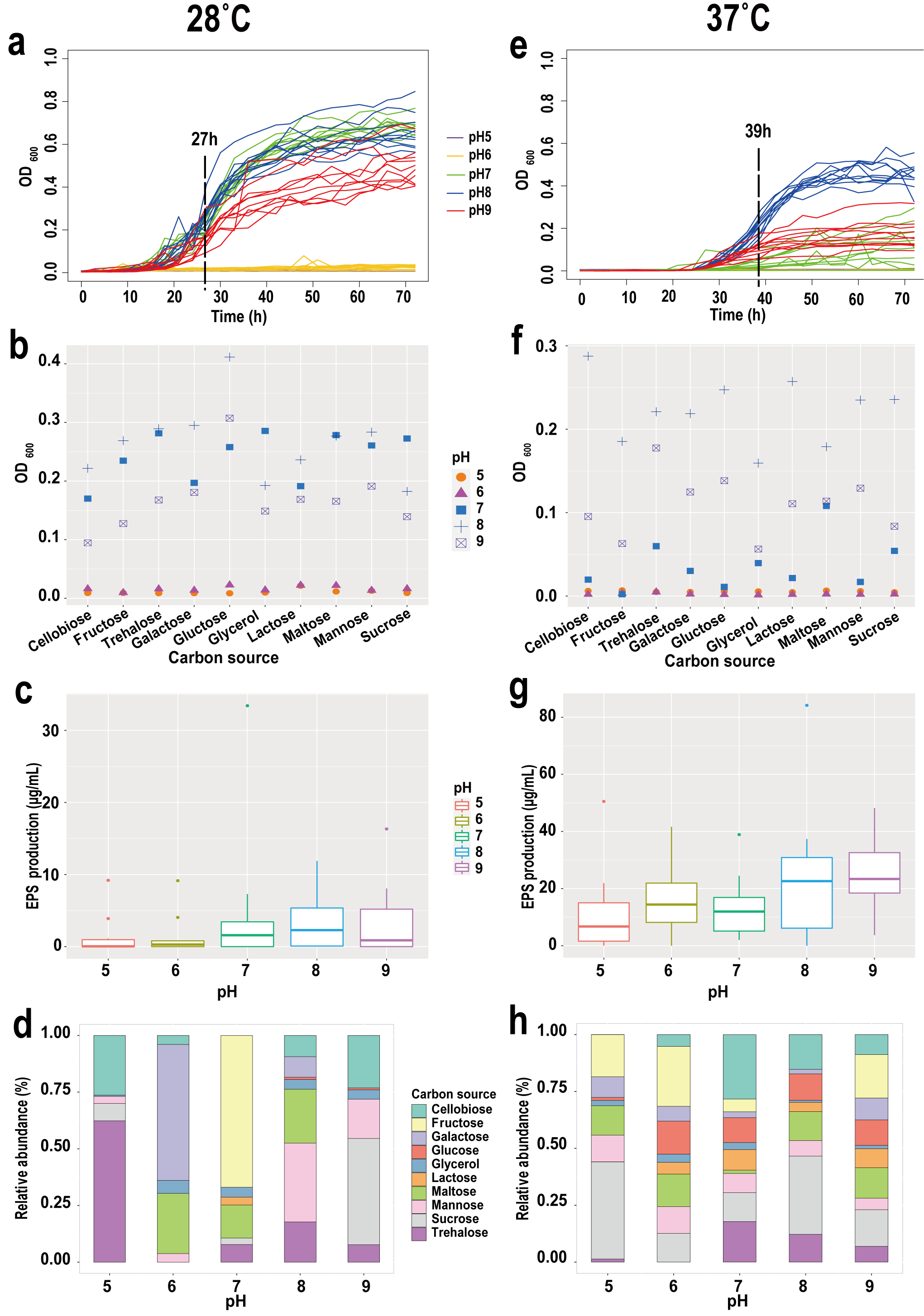
**Figure S7. Bacterial growth OD value and EPS production of AM1-D1 cultured under different conditions for 72h.** (a, e) Bacterial growth curves under different PH levels, each comprising ten carbon source conditions. Dashed lines indicate the mid-log phase time points; (b, f) OD values at the mid-log phase time points across 50 distinct culture conditions; (c, g) EPS production (μg/mL) after 72 hours of fermentation. Different colored box plots correspond to different pH levels, with each pH group containing ten carbon source conditions; (d, h) Relative EPS yield per carbon source at each pH level after 72 hours of fermentation. Panels (a), (b), (c), and (d) present data measured at 28 °C, while panels (e), (f), (g), and (h) present data measured at 37 °C.

**
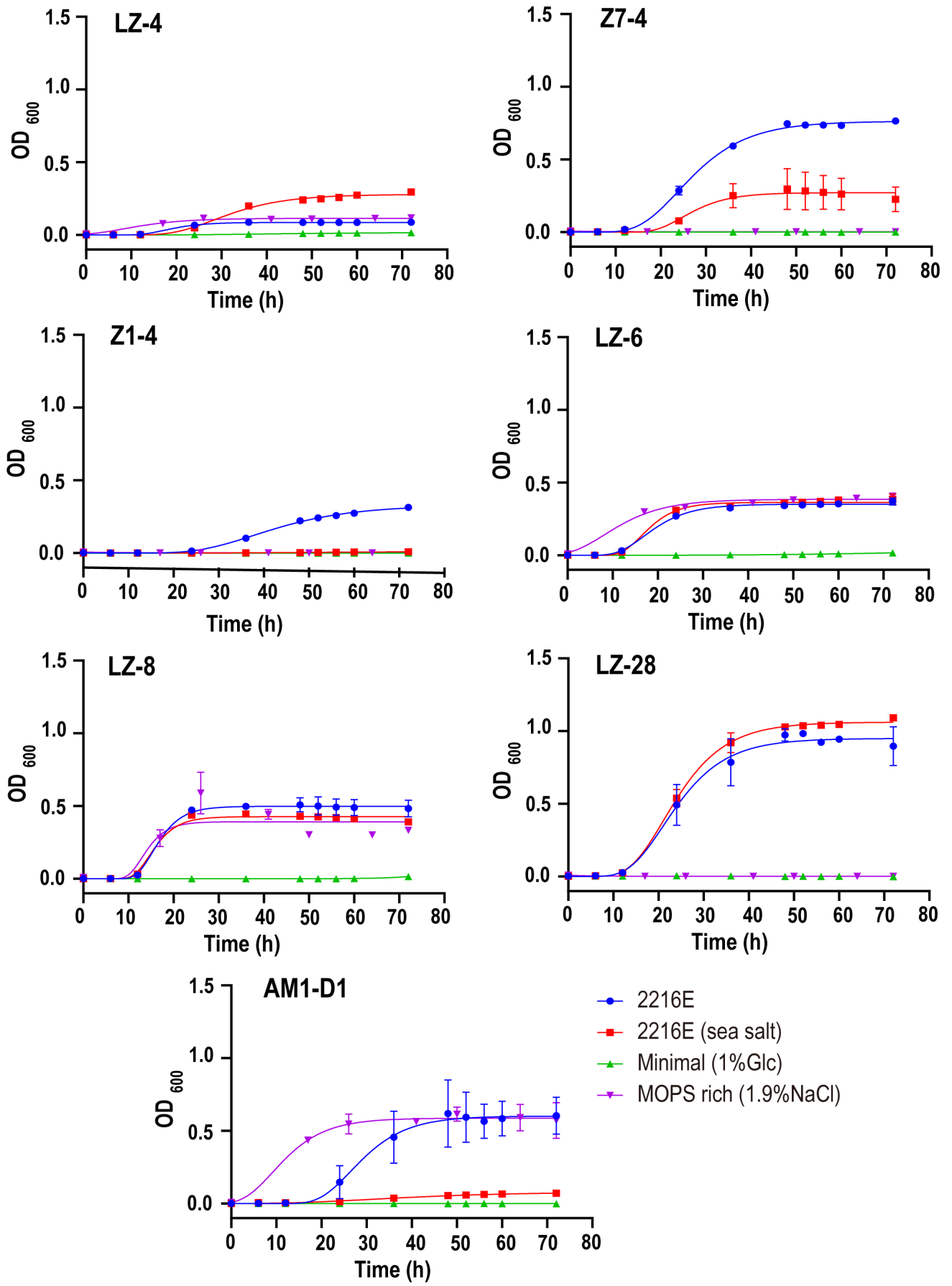
Figure S8. Determination of bacterial growth in four kinds of media.** The x-axis indicates the incubation time (up to 72 hours), and the y-axis shows the OD600 absorbance value of the bacterial suspension. Colored curves represent different media: blue, 2216E; red, 2216E (sea salt); green, Minimal medium with 1% glucose (Glc); and purple, MOPS rich medium with 1.9% NaCl. The OD value at each point on the growth curve is the average of three independent replicate experiments.

**
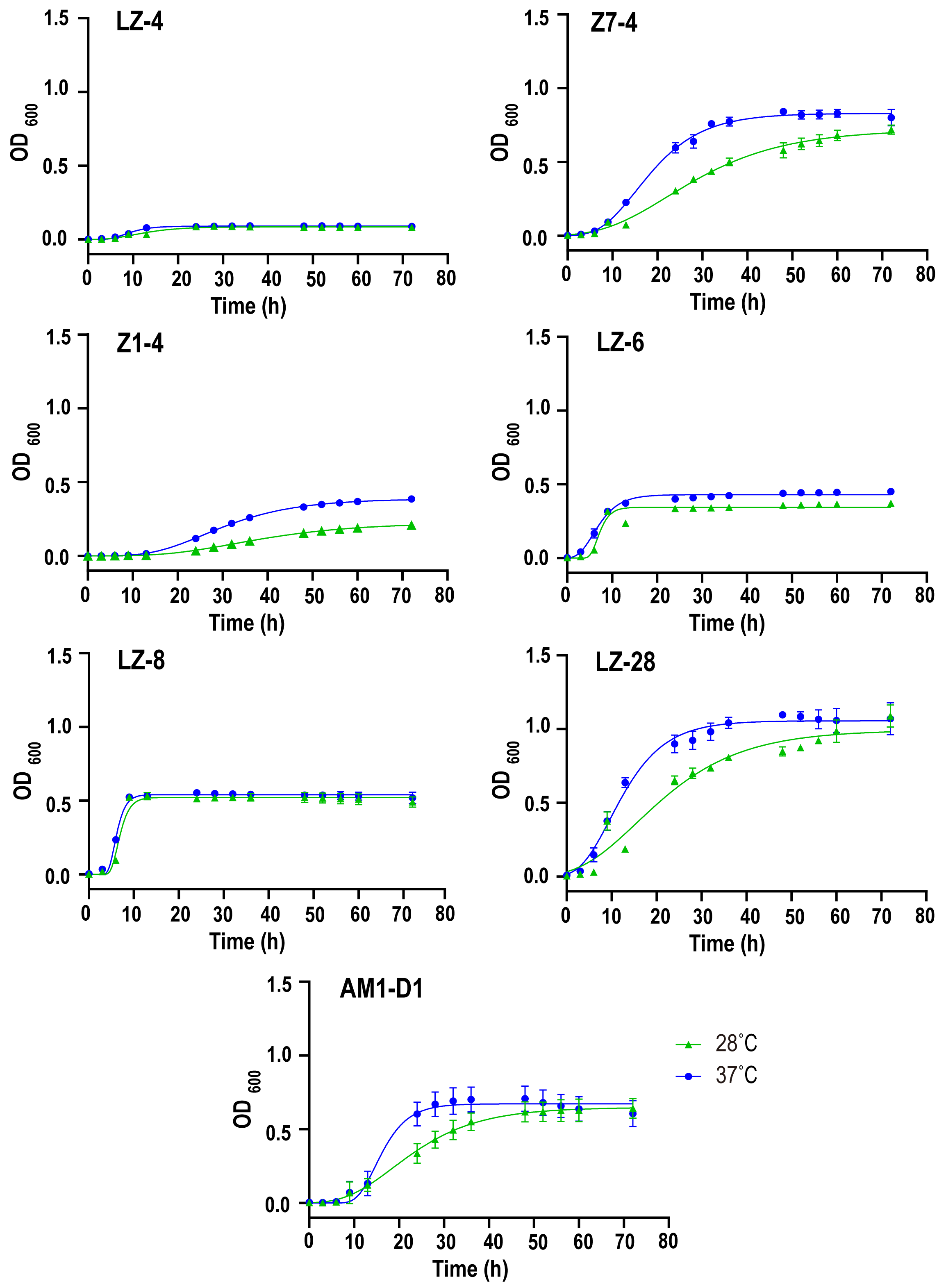
Figure S9. Determination of bacterial growth at two temperatures.** The x-axis indicates the incubation time (up to 72 hours), and the y-axis represents the OD600 absorbance value of the bacterial suspension. The color of the curves indicates the growth temperature: green for 28 °C and blue for 37 °C. The OD value at each point on the growth curve is the average of three independent replicate experiments.

**
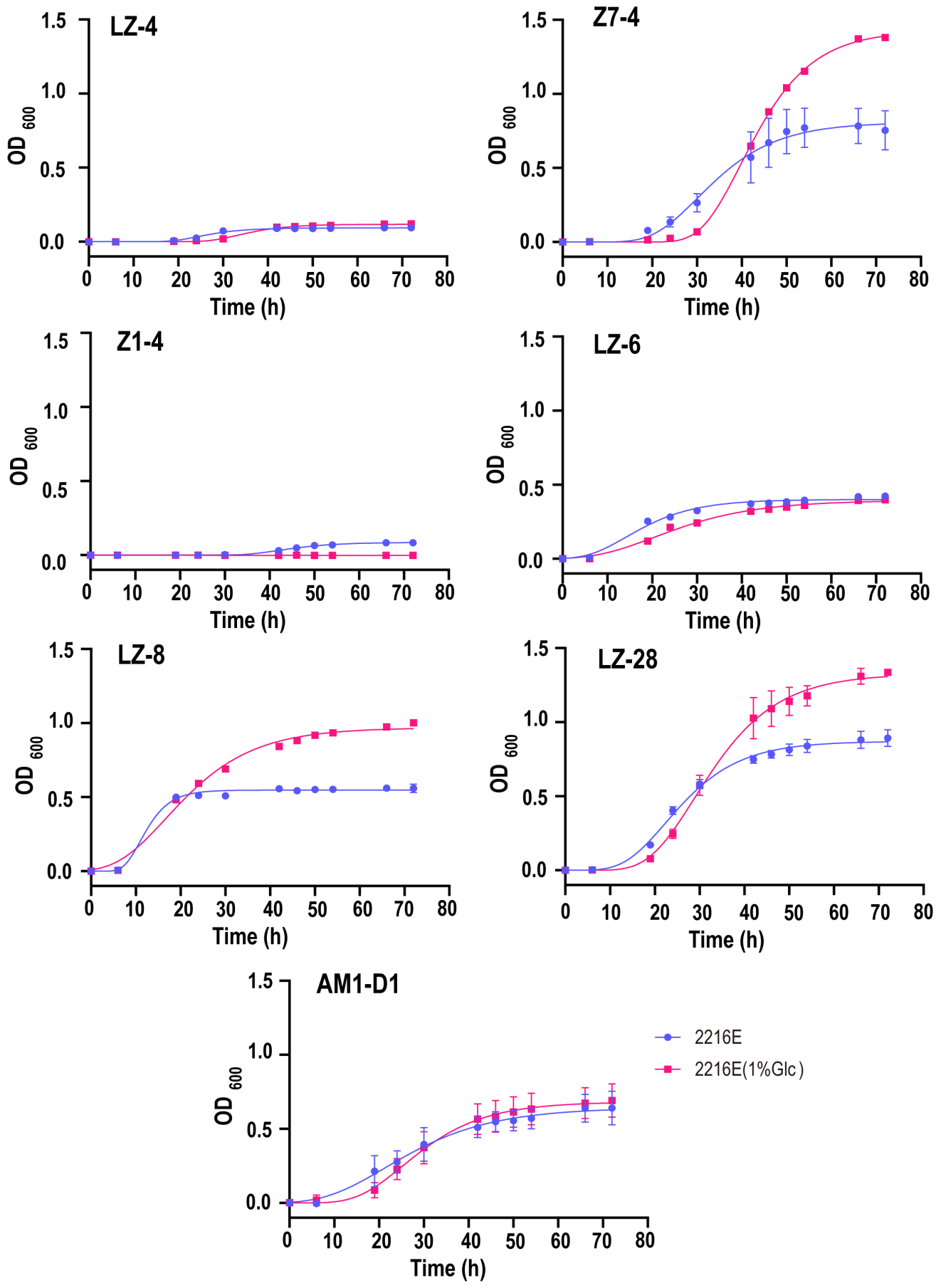
Figure S10. Determination of bacterial growth with additional carbon source in medium.** The x-axis represents time, with the measurement endpoint at 72 hours, and the y-axis represents the OD600 absorbance value of the bacterial suspension. The color of the curves indicates the type of medium used: light purple for 2216E and rose red for 2216E (1% Glc). The OD value at each point on the growth curve is the average of three independent replicate experiments.

**
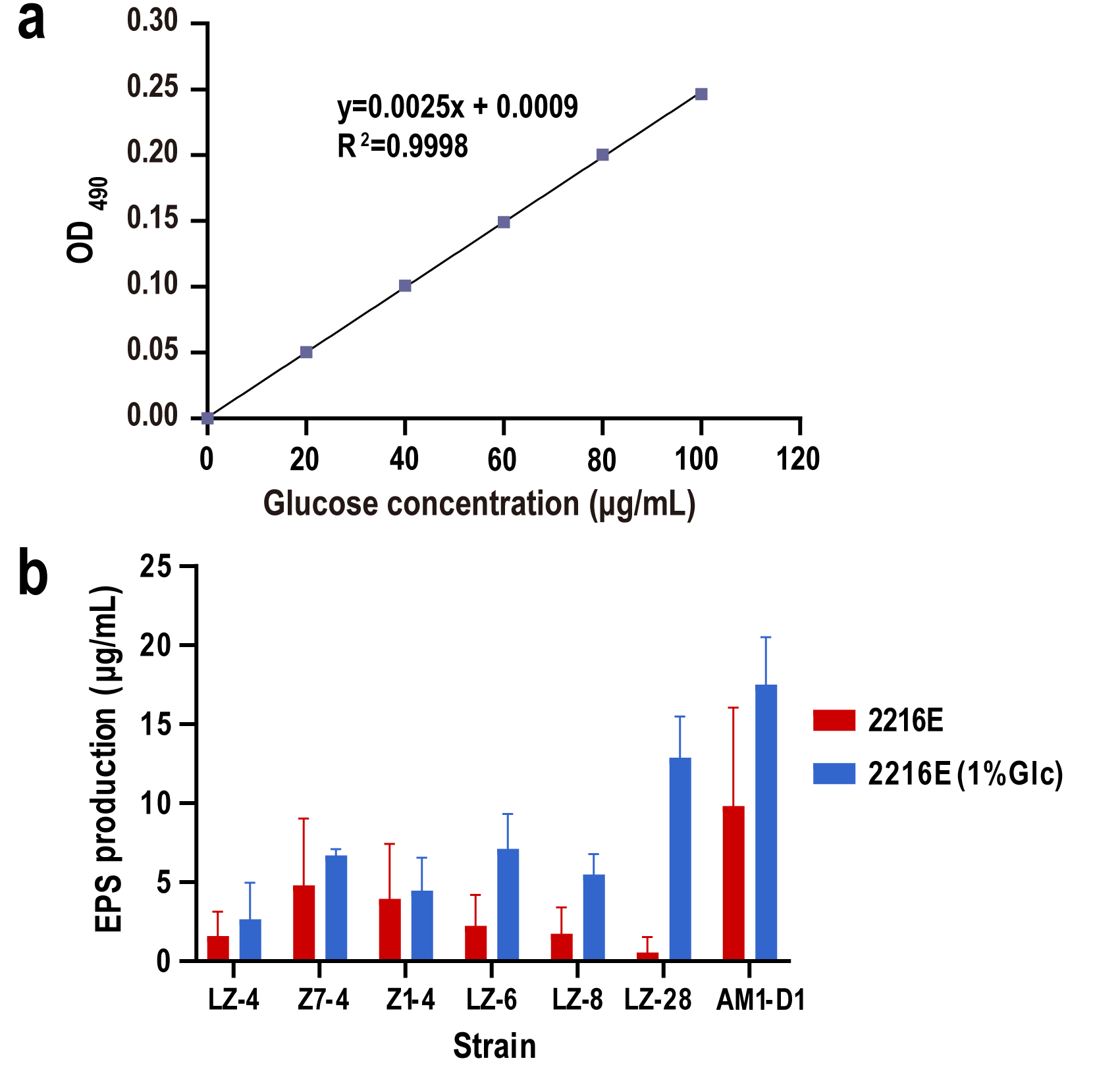
Figure S11: Detection of EPS concentration with or without extra carbon source.** (a) The standard curve for glucose concentration, with the x-axis representing sugar concentration (μg/mL) and the y-axis representing the OD490 absorbance value; (b) The EPS production by seven marine bacterial strains, with x-axis showing the strain names and the y-axis showing EPS yield (μg/mL). Red bars represent cultures grown in 2216E medium, while blue bars represent cultures grown in 2216E supplemented with 1% glucose (Glc).
